# Molecular Morphology and Function of Stromal Cells

**DOI:** 10.3390/ijms222413422

**Published:** 2021-12-14

**Authors:** Mirko Manetti

**Affiliations:** Department of Experimental and Clinical Medicine, Section of Anatomy and Histology, University of Florence, 50134 Florence, Italy; mirko.manetti@unifi.it

The term “stromal cells” refers to a highly heterogeneous class of connective tissue cells that build the infrastructure of any organ and fulfill a variety of fundamental roles in health and disease. Embedded into the framework of stromal cells are parenchymal cells, which define the specific function of an organ. Distinctive populations of stromal cells with different morphologies and functions include (i) fibroblasts, pericytes, and telocytes that are widely distributed throughout various organ systems; (ii) cells with stemness features, such as bone-marrow-derived mesenchymal stem/stromal cells (MSCs) and adipose tissue-derived stem/stromal cells (ASCs); and (iii) stromal cells specifically restricted to some organs, such as interstitial cells of Cajal in the gastrointestinal tract muscularis and fibroblastic reticular cells in secondary lymphoid organs, among others.

In recent years, there have been substantial advances in our understanding of stromal cell biology, especially the molecular signals underlying their contribution to several biological processes in different tissues, including tissue morphogenesis and development, maintenance of local tissue homeostasis, tissue injury, regeneration, immune responses, cancer, and other pathologies. Increasing evidence indicates that stromal cells are leading actors in shaping the organization, integrity, and dynamics of their own microenvironment, but their phenotype and functions are tightly dependent on the specific tissue microenvironment wherein they reside. Expanding our knowledge of the cellular and molecular mechanisms regulating the interactions of stromal cells among themselves and with neighbor parenchymal cells or tissue-resident stem cells in physiological and pathophysiological contexts is crucial to gain insights into their potential clinical relevance as therapeutic targets or tools in the translational field of tissue engineering and regenerative medicine.

In such a scenario, the collected contributions of this Special Issue of the *International Journal of Molecular Sciences*, “Molecular Morphology and Function of Stromal Cells”, consisting of seven original articles and three reviews, cover different aspects of stromal cell biology/pathobiology, including the role of the stromal microenvironment and stromal cells in diseases and novel therapeutic strategies.

In this collection, Acar and colleagues [1] present very interesting results of comparative proteome analysis of Muse stem cells vs. MSCs and in vitro-generated pluripotent stem cells (iPSCs) under basic conditions and following oxidative stress, which allowed the identification of the specific ontologies, pathways, and networks related to the characteristic enduring capacity of these adult stem cells to cope with endogenous and exogenous genotoxic stress. Indeed, Muse cells showed to be enriched in stress response-related endosomal vacuolar trafficking, ubiquitin and proteasome degradation, and reactive oxygen scavenging pathways, and showed a protein–protein interacting network with two key nodes displaying a high connectivity degree and betweenness, namely NFKB and CRKL [1]. Overall, this study represents an important step forward in the understanding of the molecular mechanisms underlying the peculiar stress resistance of Muse cells that, indeed, have been reported to survive in adverse microenvironments, such as those present in damaged/injured tissues [1]. Thus, these findings strengthen the idea that Muse cells, which are present in the stroma of several organs, may represent a valid natural alternative to MSCs and iPSCs for regenerative medicine purposes [1].

In another original study with a similar approach, Aasebø and co-authors [2] performed a global proteomic profile comparison of bone-marrow-derived osteoblasts and MSCs, whose targeting is currently regarded as a possible strategy for the treatment of malignant or non-malignant bone diseases, such as osteoporosis. The authors found a large overlap in the profiles for the two cell types, which was not surprising as MSCs can differentiate into osteoblasts [2]. Interestingly, osteoblasts displayed specific proteins, including several extracellular matrix proteins and a network of proteins that influence intracellular signaling (Wnt/Notch/Bone morphogenic protein pathways) and bone mineralization, which suggests that targeting of these proteins will possibly have minor effects on MSCs [2]. Moreover, the osteoblast and MSC proteomic profiles were found to be significantly altered by culture in serum-free media, thus highlighting the need for a careful standardization of the ex vivo handling of these cells for clinical medicine applications [2].

The original contribution of Squecco et al. [3] demonstrated the potential of bone-marrow-derived MSC secretome in relieving experimental skeletal muscle damage induced by ex vivo eccentric contraction. Of note, the MSC secretome was found to attenuate the eccentric-contraction-induced muscle tissue structural damage and alterations of sarcolemnic functional properties by protecting myofibers from apoptosis and likely contributing to the replenishment of the muscle resident myogenic satellite cell reservoir [3]. Indeed, the MSC secretome was able to affect the functionality of satellite cells and the distribution of telocytes/CD34^+^ stromal cells, a distinctive stromal cell type thought to play an important “nursing” role for these myogenic cells [3]. Collectively, these promising experimental findings provide the necessary groundwork for further investigation of the feasibility and usefulness of MSC secretome local administration to treat skeletal muscle injuries [3].

The in vivo study by Rocha and colleagues [4] reports the effectiveness of aquatic training as a rehabilitation method after joint immobilization to reduce skeletal muscle atrophy and promote regenerative processes in the myotendinous junction microenvironment, possibly involving tissue-resident telocytes/CD34^+^ stromal cells.

Díaz-Flores and colleagues contributed to this Special Issue with one original paper [5] and one review [6], both focused on telocytes/CD34^+^ stromal cells. In the original work, telocytes/CD34^+^ stromal cells were revealed as an important, though previously neglected, source of cancer-associated fibroblasts in invasive lobular carcinoma of the breast [5]. The review article points to the role of telocytes/CD34^+^ stromal cells in skin pathology [6]. Telocytes/CD34^+^ stromal cells were analyzed in normal skin tissue, and their distribution was compared with that observed in different kinds of skin lesions, including tumor or tumor-like conditions and non-tumoral skin pathologies, such as fibrosing/sclerosing diseases, basophilic degeneration of collagen, dermatitis, vasculitis, folliculitis, and perifolliculitis, among others [6]. Telocytes/CD34^+^ stromal cells are still a poorly characterized cell type because of their relatively recent identification, and, hence, these contributions pave the way for further research addressing the possible implication of these peculiar cells in other pathophysiological processes.

A comprehensive review written by Rautiainen et al. [7] is dedicated to the angiogenic effects and crosstalk of ASCs and their extracellular vesicles with endothelial cells. Focusing on different co-culture systems and co-transplantation studies between ASCs and endothelial cells, the authors review the state-of-the-art evidence for the ability of ASCs to differentiate into endothelial cells and pericyte-like cells and to secrete angiogenesis-promoting growth factors and extracellular vesicles, which makes them an ideal option in cell therapy and regenerative medicine approaches for conditions including tissue ischemia [7].

In the field of tissue engineering for regenerative-medicine purposes, Martinez-Garcia and colleagues [8] investigated the combination of gelatine methacryloyl hydrogels and ASCs to model stem cell-driven repair and to understand cell–biomaterial interactions in 3D environments. Indeed, within a 3D environment, stromal cells experience biochemical and biomechanical stimuli, with the extracellular matrix regulating cellular behavior via activation of intracellular signaling pathways and, in response, cells degrade, deposit, and rearrange matrix components. The authors found that ASCs transform gelatine methacryloyl hydrogel viscoelasticity into a more fluid environment and alter the hydrogel mechanical stability [8]. In addition, out of the concentrations studied, 5% gelatine methacryloyl hydrogel provided the most conducive biomechanical environment for ASCs to spread and remain viable [8].

Through in vivo research, Lu et al. [9] disclose, for the first time, the important role of the FOXO1/β2-AR/p-NF-κB p65 pathway in the development of endometrial stromal cells in pregnant mice under restraint stress, providing a scientific basis for the origin of psychological stress in pregnant women.

Finally, the review by Sadeghi et al. gives an overview of the signaling pathways that regulate lymphoma cell migration and interaction with stromal cells within the tumor microenvironment, with a focus on Mantle cell lymphoma, and highlights the fundamental role of epigenetic mechanisms in integrating signals at the level of gene expression throughout the genome [10].

In aggregate, the contributions published within the Special Issue “Molecular Morphology and Function of Stromal Cells” are excellent examples of the recent advances made in the broad field of stromal cell research and will hopefully open new avenues for the exploration of stromal cell pathobiology, providing important discoveries into novel therapeutic approaches for different kinds of diseases. I thank all the authors and reviewers for their valuable work and the time dedicated to providing these excellent contributions to this Special Issue of the journal. I also acknowledge Ansel Liu and the *International Journal of Molecular Sciences* Editorial Office for their professional work in making this Special Issue successful. A “Molecular Morphology and Function of Stromal Cells 2.0” Special Issue is now open for submission of original manuscripts and reviews authored by outstanding experts dealing with any aspects of stromal cell biology.
